# Lower-Limb Perfusion and Cardiovascular Physiology Are Significantly Improved in Non-Healthy Aged Adults by Regular Home-Based Physical Activities—An Exploratory Study

**DOI:** 10.3390/life14020241

**Published:** 2024-02-08

**Authors:** Margarida Florindo, João Gregório, Luís Monteiro Rodrigues

**Affiliations:** 1 CBIOS—Research Center for Biosciences & Health Technologies, Universidade Lusófona, 1749 024 Lisboa, Portugal; mflorindo@esscvp.eu (M.F.);; 2 Department of Physiotherapy, ESSCVP—Portuguese Red Cross Health School, 1300 125 Lisboa, Portugal; 3 Escuela de Doctorado, Programa de Ciencias de la Salud, Universidade de Alcalá, 28034 Madrid, Spain

**Keywords:** physical activity, home health, non-healthy aged adults, cardiovascular physiology, distal perfusion, mean arterial pressure

## Abstract

Common daily activities including walking might be used to improve cardiovascular health in the presence of disease. Thus, we designed a specific home-based physical activity program to assess cardiovascular indicators in an older, non-active, non-healthy population. Ten participants, with a mean age of 62.4 ± 5.6 years old, were chosen and evaluated twice—upon inclusion (D0), and on day 30 (D30)—following program application. Perfusion was measured in both feet by laser Doppler flowmetry (LDF) and by polarised spectroscopy (PSp). Measurements were taken at baseline (Phase 1) immediately after performing the selected activities (Phase 2) and during recovery (Phase 3). Comparison outcomes between D0 and D30 revealed relevant differences in Phase 1 recordings, namely a significant increase in LDF perfusion (*p* = 0.005) and a significant decrease in systolic blood pressure (*p* = 0.008) and mean arterial pressure (MAP) (*p* = 0.037). A correlation between the increase in perfusion and the weekly activity time was found (*p* = 0.043). No differences were found in Phase 2, but, in Phase 3, LDF values were still significantly higher in D30 compared with D0. These simple activities, regularly executed with minimal supervision, significantly improved the lower-limb perfusion while reducing participants’ systolic pressure and MAP, taken as an important improvement in their cardiovascular status.

## 1. Introduction

Healthy lifestyles are central to longer and healthier lives [1,2]. A growing number of papers, including systematic reviews, tend to demonstrate that physical activity, defined as any body movement resulting in an increase in energy expenditure [3,4], is particularly beneficial to improve global cardio-circulatory function, even in the presence of age-related comorbidities or after serious events such as cancer or stroke [2,5,6,7].

Our group has dedicated special attention to studying the impact of movement on lower-limb skin perfusion. Many pathologies converge to this distal region of the circulatory system, eventually leading to or facilitating various processes, some of which are life-threatening [8,9]. Thus, lower-limb skin perfusion is a viable marker of vascular health. We have demonstrated that short-duration, low-intensity activities such as walking and short-duration passive interventions such as single-limb massage significantly improve both lower-limb perfusion and the individual’s global cardiocirculatory conditions [10,11] in normal (non-disease) participants. Other studies also have shown that short-duration and low-intensity exercises, such as isometric plantar flexion [12,13], “step-in-place” [13,14], or unipodal flexion [15], have similar effects, improving the individual’s distal perfusion as well as general haemodynamics [10,16]. These promising results led us to consider these common activities as potential instruments to promote health at home in older, non-healthy, non-active, and less compliant populations. Home-based health programs are not a novelty regarding conventional supervised therapeutics, although systematic reviews are scarce and primarily focused on specific conditions [17,18,19,20]. Nevertheless, several studies and trials have confirmed the interest and effectiveness of home-based health programs in cardiovascular rehabilitation [7,21,22,23,24].

Based on our previous experience, we conceived the present preliminary study to determine the cardiovascular impact, if any, of a simple, easy to manage (minimal supervision), home-based intervention involving common day-to-day activities, previously tested and now applied to a pre-selected cohort of older, non-healthy participants with multiple co-morbidities. A secondary objective focused on participants’ perceptions of the importance of potential benefits in their daily habits and how this program might affect their motivation and expectations.

## 2. Materials and Methods

### 2.1. Participants

This study involved a convenience sample of twelve participants of both sexes (five women and seven men) enrolled from the clinical practice of one of the authors according to pre-defined inclusion/non-inclusion criteria. The main criteria were being older than fifty years, diagnosed with one or more age-related co-morbidities such as overweight–obesity, hypertension, dyslipidaemia, and diabetes, and being regularly medicated. A sedentary lifestyle pattern as described by the WHO (World Health Organisation) [1] was also considered as an inclusion criterion. The self-reported physical activity of participants (less than 150 min of moderate physical activity per week, or less than 5000 steps per day, as recorded by smartphone) confirmed this pattern. The “Physical Activity Enjoyment Scale (PACES)” questionnaire validated for the Portuguese population [25,26] was used. One of the participants could not be classified as sedentary (according to reported activities), and another participant interrupted the protocol in the second week due to frequent travel. These participants were excluded from the study. The final convenience sample included ten individuals (Table 1) with a mean age of 62.4 ± 5.6 years (five women, 62.6 ± 8.3 years old and five men, 62.2 ± 1.3 years old). Due to the preliminary character of the study, participants served as their own controls.

The characterisation of participants also included the calculation of the body mass index (BMI) [27] and various other markers, e.g., the ankle–brachial index (ABI) [28], blood pressure (systolic, diastolic) and calculated mean arterial pressure (MAP), obtained by an oscillometer method.

The study followed all principles of good clinical practice according to the World Medical Association’s Declaration of Helsinki recommendations and subsequent amendments [29], including informed consent obtained from all subjects prior to the initiation of the study. The study protocol was previously approved by the Universidade Lusofona’s Health Science Ethics Committee (EC. ECTS/P03.20).

### 2.2. Experimental Setting

All biometrical measurements were taken in a laboratory and were performed by the same operators. Participants were allowed to adapt to room conditions for 20–25 min (mean temperature 24 ± 1 °C, 54 ± 2% humidity, and controlled light) prior to measurements.

The ankle and arm arterial pressures were measured with a digital sphygmomanometer (Pic 22012000200 Sphygm Classic Check; Artsana S.p.A., Como, Italy), in the supine position, to calculate the ABI by the ankle systolic pressure/brachial systolic pressure ratio [28]. Related variables were registered in the standing position, such as systolic blood pressure (SYS_P), diastolic blood pressure (DIAS_P), mean blood pressure (MAP), and pulse rate (PR). Perfusion measurements were taken after ten minutes in the standing position.

Two laser Doppler flowmetry probes (LDF—Perimed PF5010 System; Perimed, Stockholm, Sweden) were applied to measure distal skin perfusion (PU, reported in arbitrary units) in each foot. This optical technology quantifies the movement of red blood cells at a depth of approximately 0.5 mm, using a laser beam of 780 nm [30,31]. Both LDF probes were fixed with a double-sided adhesive on the dorsal aspects of the third toe metatarsus in both feet.

The polarised light spectroscopy system (Tissue Viability Image System—TiVi701; WheelsBridge, Linkoping, Sweden) was also used to assess the skin surface perfusion. This system uses an adapted digital camera placed 30–60 cm away from the dorsal region of the forefoot. Polarised light spectroscopy (PSp) provides the concentration of red blood cells (CRBC in arbitrary units) in real time through video images of the microcirculation obtained with cross-polarisation filters. This technology allows perfusion to be measured at different (skin) depths through the application of a proprietary algorithm in which each pixel corresponds proportionally to the perfusion dispersion in the skin tissue in the selected region of interest (ROI) [32,33].

Two experimental moments were considered—day zero (D0) and day 30 (D30). The baseline measurements of all variables on D0 was made immediately prior to the programmed activity intervention. This intervention was oriented by FITT principles (F—frequency, I—intensity, T—time/duration, and T—type of activities to perform). For each participant, we calculated the intensity involved with the program activities using the Karvonen formula [(Max Heart Rate—Resting Heart Rate) × 60% + Resting Heart Rate] [34]. These calculations confirmed that the programmed activities were classified as light intensity for every participant, dispensing the need to introduce any further indicators of activity intensity. Hence, we defined a sequence of activities requiring eleven minutes: five minutes of stepping in place with an intensity chosen by the individual, one minute of isometric plantar flexion, and five minutes of regular walking through a random circuit within the participant’s home at a slow, comfortable pace. Instructions regarding these exercises were drawn schematically on paper and provided to all participants. The program was set to be repeated daily once a day at home for thirty days.

Each experimental moment included three phases—Phase 1, corresponding to the register of basal conditions with volunteers standing for 5 min; Phase 2, corresponding to measurements following the sequential program execution; and Phase 3, in which participants returned to the standing position to recover for five minutes.

During the intervention period, participants were contacted three times a week by one researcher (by voice and/or video call) to confirm compliance and to provide clarification, as needed.

In addition, participants were given a questionnaire on D30 to identify the perception of physical activity enjoyment (PACES) [25,26] and the impact of daily physical activity in the studied weeks (PGIS Portuguese version) [26].

### 2.3. Statistical Analysis

Descriptive and comparative statistical analysis was performed using the IBM SPSS Statistics for Windows 28.0 software (IBM Corp., Armonk, NY, USA) and Jamovi 2.3.16 [35]. Intra-individual perfusion variations between the right and left feet were tested by the Wilcoxon sign-rank test. The same test was used to compare phases and to identify variations after 30 days of application of the physical activity protocol. Variations were also expressed in percentages (%), and the data representing the differences in perfusion between D0 and D30 were transformed into new variables (Delta) to explore potential correlations within the questionnaire components using the Spearman’s correlation coefficient tests. A linear regression was also applied to assess the association of perfusion with BMI, adjusting for sex. A confidence level of 95% (*p* < 0.05) was adopted.

## 3. Results

As shown in Table 1, the studied individuals shared various prevalent co-morbidities. The participants were overweight (BMI (25.6 ± 2.9), a recognised risk factor for cardiovascular disease, and had high MAP values (95.7 ± 6.5 mmHg) close to the highest limits of normal. ABI values indicated that participants had no peripheral vascular disease. Most of these participants had at least two associated co-morbidities, where overweight and dyslipidaemia were the most common (7 in 10), followed by hypertension (5 in 10). In addition, all participants were taking regular medications, which were maintained during the study.

Regarding physical activity, we observed on D0 that the number of daily steps within this group was 3610 ± 541.3, which is lower than the recommended 5000–7500 steps/day reference for the prevention of vascular impairment. The number of minutes of physical activity per week was also below the WHO recommendation for non-sedentary adults, which indicates that adults between 18 and 64 years old should practice at least 150 min of moderate activity per week.

At baseline (Phase 1 of D0), no statistically significant differences were found among participants regarding perfusion (measured with LDF and PSp) SYS_P, DIAS_P, and MAP and PR.

Table 2 shows the progression of variables as registered on D0 and D30 (Table 2). On D0, the impact of activity on perfusion in Phase 2 relative to Phase 1 was significant for the PU variable obtained from LDF (*p* < 0.05). The same observation was noted for PR, as for all the other pressure-related variables. Only PSp CRBC showed a non-significant difference after the programmed activity. On D0, lower values for SYS_P (*p* = 0.038) DIAS_P (*p* = 0.049) and MAP (0.007) were observed in Phase 3, while the pressure-related variables were slower to recover. On D30, we found that the programmed activity evoked significant differences (*p* < 0.05) in all variables in Phase 2, while promoting a faster recovery to baseline values in all variables (without exception) in Phase 3.

We also analysed the differences in these outcomes between D0 and D30 (Table 3). A significant increase in LDF perfusion values was noted in Phases 1 and 3 following the thirty-day intervention program. Significant changes in perfusion as measured by PSp were not found.

Regarding the blood pressure variables, a statistically significant decrease in SYS_P (*p* = 0.008) and MAP (*p* = 0.037) was detected.

A statistically significant correlation with the perfusion changes that occurred between D0 and D30 was also noted. Considering the difference between D0 and D30, an increase in LDF perfusion was found, while the perfusion measured by PSp decreased.

**Figure 1:**
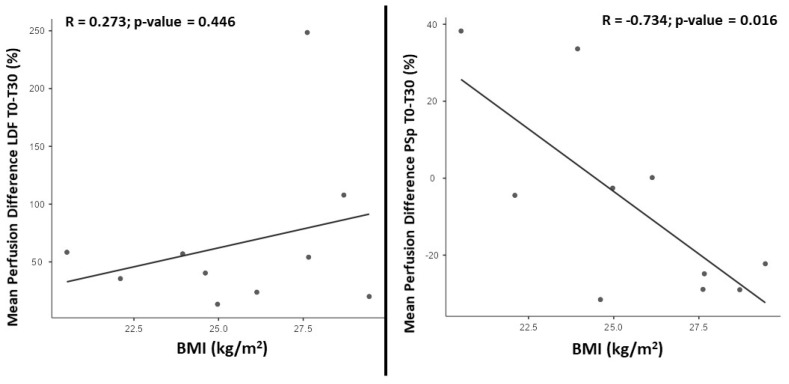
BMI and perfusion measured with LDF (**left**) and PSp (**right**) correlation (Pearson) found between D0 and D30.

The LDF perfusion differences between D0 and D30 were not associated with BMI (Figure 1—left panel). However, perfusion differences measured with PSp could be associated with the BMI (Figure 1—right panel). Using linear regression adjusting for the effect of sex, we found this association to be statistically significant (β = −0.798 [95%CI −1.320; −0.275]; *p* = 0.009).

The application on D30 of the Physical Activity Enjoyment Scale (PACES) [25] and the Patient Global Impression of Change Scale [36] (from 1 to 7) revealed median scores of 3.8 and 4.5, respectively. Lower values indicate a perception of lower importance or effect.

A correlation was found between the significant PU variations detected on D30 with questionnaire answers, which allowed us to identify strong correlations with the ease of memorising the program (*p* = 0.023), the importance the activities might represent in the participant’s health (*p* = 0.039), the promotion of better life habits (*p* = 0.041), and the feeling that the activity is interesting (*p* = 0.007). No correlations were found with the increase in the number of steps per day or the time spent standing during the day.

## 4. Discussion

Consistent results regarding the effects of common daily active movements (e.g., walking) on the lower-limb perfusion in healthy populations were our main motivation to develop this experiment. Therefore, we designed the present study as a preliminary evaluation of the utility of some of these combined activities as potential promoters of cardiovascular health in a group of older individuals with age-related co-morbidities. It is clear that light and short-term activity (with static and semi-dynamic conditions) and even posture can significantly alter the distribution and perfusion of blood in both feet, even when the challenge involves one single limb, but can also modify cardiac performance [10,11,12,13,14,15,16]. More evidence has accumulated regarding the benefits of any activity, including walking as opposed to “inactivity”—in the prevention of and rehabilitation from common cardiovascular events [23,24,37,38,39]. Therefore, knowing that ageing and a sedentary lifestyle are strong determinants of disease [6,8,40], we decided to observe the impact of a short-term, light-intensity combination of three movements previously determined to be capable of (a) encouraging autonomous walking with both ambulatory and “step in place” motion if confined for whatever reason at home, (b) requiring minimum supervision to be properly accomplished, and (c) promoting calf pump activation, which is regarded as vital to maintain proper vascular function in the lower limbs, especially in the orthostatic position, by plantar flexion [40,41,42]. The result was a very simple physical activity program involving five minutes of stepping in place, one minute of isometric plantar flexion, and five minutes of regular gait. This eleven-minute program was applied for four weeks in a home-based environment. All participants had smartphones with an application capable of recording the number of steps taken each day. This step register was checked at day zero and then again at day thirty.

As shown in Table 2 and Table 3, this simple program led to significant modifications in the cardiovascular physiology of these participants. On Day 0, the sequence of exercises clearly changed the perfusion variables measured by LDF but not those measured by PsP (Table 2 compares Phase 2 with Phase 1; see discussion further ahead). All other haemodynamic variables changed in the same direction as a direct result of the activity involving significant increases in blood pressure, MAP, and PR. In Phase 3, perfusion and PR rapidly returned to baseline levels, but other haemodynamic markers remained significantly altered by the end of the experiments (Table 2 compares Phase 3 with Phase 1). Similar comparisons obtained from the (same) participants’ outcomes on D30 after the intervention enable the better characterisation of this reality (Table 3).

As is shown, all changes evoked by the exercise itself were similar in all variables (Table 3 compares D0 and D30 in Phase 2). However, baseline recordings of these outcomes were clearly different between D0 and D30, displaying a significant improvement in baseline perfusion detected by LDF and a significant decrease in SYS P and MAP (Table 3 compares D0 and D30 in Phase 1). Improvements in baseline perfusion blood pressure and MAP surely involve global blood flow changes expected to be related to muscular and postural tonicity modifications [2,43,44,45]. Noteworthy is the significant reduction in blood pressure, specifically the registered decrease in SYS P in all patients. Systolic blood pressure is recognised as the most important preventable blood pressure-related risk indicator for cardiovascular disease [46,47,48,49], and MAP is recognised as an important marker directly related to the cardiovascular and cerebrovascular risk of stroke [43,44,48]. Finally, we perceived that pos-activity recovery was much faster on D30, as we could not find any significant differences in any variable except for LDF perfusion (Table 3 compares D0 and D30 in Phase 3). The absence of differences in haemodynamic variables in Phase 3 suggests a more effective cardiovascular adaptation to the challenger (of exercise) when compared with D0. These differences between D0 and D30, especially at baseline, might be due to the adaptive process evoked by a regular increase in activity.

All LDF registers involved the simultaneous recording of skin temperature, which showed no variation between D0 and D30. On D30, the participants reported no relevant changes in ongoing medication, their nutritional habits, or body mass during this period.

A significant increase in LDF perfusion values was noted in Phase 1 and 3 following the thirty-day intervention program, not followed by PSp measurements. LDF and PsP provide different perfusion measurements related to their biophysical principles and the particular structure of skin vascular organisation [11,48,50]. Although sharing the same optical basis, LDF is a single-point measurement technology using a 780 nm laser light that deeply penetrates the skin to 0.6 mm, while PsP uses polarised light scattered on a more superficial and wider skin surface [32,33]. Different light penetration means different interactions between the light and the tissue [32]. From the structural perspective, the particular organisation of skin microcirculation with two vascular plexuses (superficial and deep) involving vessels with different dimensions and amounts of blood also implies, as recently demonstrated, that blood travels between these two plexuses during the microcirculatory adaptation to acute changes [50,51]. The results suggest that higher LDF values reflect higher amounts of blood in the deeper structures assessed by LDF light, surely related to the activity interventions and increased requirements for blood within the muscle.

Adults with higher BMIs showed a larger reduction in PSp perfusion after following the program for 30 days (Figure 1), in line with previously published observations regarding the relationship between BMI and perfusion [52].

As a final element to our study, we questioned our cohort regarding their opinion about potential lifestyle modification and how this program could be considered as an instrument of positive change. Follow-up was indicated as key for program compliance. Participants also indicated that the characteristics of being “easy to memorise and execute”, “simple to develop”, and needing “no specialised supervision” were important for this home-based application purpose.

Therefore, under the present experimental conditions, assuming the exploratory nature of these results, we propose the following:

- This set of common activities, previously studied in healthy cohorts, might be used in a home-health program to improve the cardiovascular status and the general health conditions of older, non-healthy, sedentary patients.
- This FITT-inspired sequence favoured the lower-limb distal perfusion and systemic haemodynamics of all patients, as detected by the consistent improvement in LDF perfusion and the reduction in systolic blood pressure and MAP.
- These impacts might be attributed to the program intervention since (a) intra-individual related features (such as skin temperature, body mass, and the fact that the patients’ medication(s) did not change from D0 to D30), (b) interindividual differences involving the fulfilment of the program were not anticipated, considering the low intensity level of activities involved, and (c) data reliability was fully ensured by the experience of the operator with the measurement technologies and methods applied here.

Nevertheless, some objective limitations should be specified as follows: (i) the dimension of the convenience sample limited the power of the analysis, and the absence of a control group was an assumed option based on the objectives of the study and our previous experience with these challenges in healthy cohorts; (ii) the limited duration of the study did not allow the establishment of the onset of significant changes, including the long-term impact on the actual level of physical fitness nor their durability; (iii) the heterogeneity of co-morbidities in the studied participants, which impairs an objective evaluation of different impacts for different disease statuses; and (iv) the presence of ongoing medications in all patients potentially influencing some of the results.

## 5. Conclusions

This home-health program, although exploratory, resulted in a significant and clinically meaningful improvement in perfusion and global haemodynamic markers in this multi-co-morbidity sedentary cohort. This type of approach to support and improve vascular health should be further explored.

## Figures and Tables

**Table 1:** Characterisation of participants at inclusion (D0). Where appropriate, results are presented as mean and standard deviation (mean ± sd). Co-morbidities were previously diagnosed by a family doctor and or specialty physicians, and all related medications, generally identified here, were unchanged during the study.

D0	Participants	
N	10	
Age, years	62.4 ± 5.6	
BMI, kg/m^2^	25.6 ± 2.9	
ABI	1.1 ± 0.1	
MAP, mmHg	95.7 ± 6.5	
Steps/day (number)	3400.5 ± 826.7	
Activity(h)/week	87.0 ± 12.3	
Lower-limb pain (VAS)	1.7 ± 1.1	
Identified Comorbidities		Identified comedication
Pre-diabetes n (%)	3 (30)	▪ no specific medicines (n = 3) ▪ nutritional control (n = 2)
Hypertension n (%)	5 (50)	▪ combination beta-blocker + ACE inhibitor/Calcium channel inhibitor (n = 2) ▪ platelet anti-aggregant (n = 2) ▪ ARA (n = 1)
Dyslipidaemia n (%)	6(60)	▪ Statins (n = 6)
Overweight n (%)	7 (60)	▪ No specific medicines (n = 3)
Two or more in the same individual n (%)	9 (90)	not applicable

BMI, body mass index; ABI, ankle–brachial index; MAP, mean arterial pressure; VAS, visual analogic scale. ACE, angiotensin-converting enzyme; ARA, angiotensin receptor antagonist.

**Table 2:** Impact of the programmed activity intervention detected at the beginning of the study (D0) and after thirty consecutive days of activity (D30). Results referred to the chosen perfusion-related variables of interest, presented as medians and Q1–Q3 (25th empirical quartile–75th empirical quartile) (see text). Statistical analysis was made with the Wilcoxon sign-rank test, * *p* ≤ 0.05.

Baseline Variables (D0)		Phase 1	Phase 2	Phase 1–Phase 2 *p*-Value	Phase 3	Phase 1–Phase 3 *p*-Value
LDF PU (AU)	med (Q1–Q3)	7.7 (6.2–10.4)	12.9 (9.0–19.9)	0.005 *	8.3 (7.4–11.0)	0.093
PSp CRBC (AU)	med (Q1–Q3)	117.8 (103.4–127.7)	111.0 (104.1–122.3)	0.169	113.4 (99.0–122.3)	0.415
SYS_P (mmHg)	med (Q1–Q3)	127.5 (118.8–130.8)	136.0 (124.5–138.0)	0.005 *	121.0 (111.5–130.3)	0.038 *
DIAS_P (mmHg)	med (Q1–Q3)	80.5 (76.0–82.5)	82.5 (77.5–85.0)	0.011 *	77.0 (73.5–82.3)	0.049 *
MAP (mmHg)	med (Q1–Q3)	95.8 (89.8–98.8)	99.5 (95.4–103.2)	0.005 *	90.0 (87.1–95.3)	0.007 *
PR (bpm)	med (Q1–Q3)	73.0 (72.3–76.3)	83.0 (77.5–91.3)	0.005 *	73.5 (69.0–79.8)	0.266
**Day Thirty (D30)**		**Phase 1**	**Phase 2**	**Phase 1–Phase 2** ***p*-Value**	**Phase 3**	**Phase 1–Phase 3** ***p*-Value**
LDF PU (AU)	med (Q1–Q3)	13.2 (11.6–15.7)	19.0 (15.7–19.5)	0.022 *	13.0 (9.9–14.7)	0.475
PSp CRBC (AU)	med (Q1–Q3)	97.7 (91.8–121.4)	119.9 (99.2–127.6)	0.037 *	101.6 (91.0–119.9)	0.721
SYS_P (mmHg)	med (Q1–Q3)	120.0 (114.8–124.8)	127.5 (125.5–133.0)	0.012 *	121.0 (116.0–126.3)	0.282
DIAS_P (mmHg)	med (Q1–Q3)	77.5 (76–81.3)	82.0 (79.3–84.5)	0.011 *	76.5 (75.0–79.3)	0.673
MAP (mmHg)	med (Q1–Q3)	92.5 (88.9–95.6)	97.3 (96.0–99.1)	0.005 *	90.8 (87.7–93.5)	0.677
PR (bpm)	med (Q1–Q3)	73.5 (68.3–76.8)	80.5 (75.0–93.5)	0.005 *	74.5 (69.0–77.8)	0.256

LDF PU, perfusion obtained with laser Doppler flowmetry; PSp CRBC, concentration of red blood cells obtained with polarised spectroscopy; AU, arbitrary units; SYS_P, systolic pressure; DIAS_P, diastolic pressure; MAP, mean arterial pressure; PR, pulse rate; bpm, beat per minute.

**Table 3:** Outcomes obtained as a result of a thirty-day daily intervention involving a programmed home-based physical activity (see text). Results presented as medians and Q1–Q3 (25th empirical quartile–75th empirical quartile). Changes are shown as a percentage (%) variation between D0 and D30 and compared with Wilcoxon sign-rank test, (* *p* ≤ 0.05).

			D0	D30	% (Mean)	*p*-Value
Phase 1	LDF PU (AU)	med (Q1–Q3)	7.7 (6.2–10.4)	13.2 (11.6–15.7)	57.3	0.005 *
	PSp CRBC (AU)	med (Q1–Q3)	117.8 (103.4–127.7)	97.7 (91.8–121.4)	(-) 7.9	0.445
	SYS_P (mmHg)	med (Q1–Q3)	127.5 (118.8–130.8)	120.0 (114.8–124.8)	(-) 5.7	0.008 *
	DIAS_P (mmHg)	med (Q1–Q3)	80.5 (76.0–82.5)	77.5 (76–81.3)	(-) 3.0	0.137
	MAP (mmHg)	med (Q1–Q3)	95.8 (89.8–98.8)	92.5 (88.9–95.6)	(-) 4.2	0.037 *
	PR (bpm)	med (Q1–Q3)	73.0 (72.3–76.3)	73.5 (68.3–76.8)	(-) 1.0	0.888
Phase 2	LDF PU (AU)	med (Q1–Q3)	12.9 (9.0–19.9)	19.0 (15.7–19.5)	25.7	0.169
	PSp CRBC (AU)	med (Q1–Q3)	111.0 (104.1–122.3)	119.9 (99.2–127.6)	8.0	0.646
	SYS_P (mmHg)	med (Q1–Q3)	136.0 (124.5–138.0)	127.5 (125.5–133.0)	(-) 3.9	0.139
	DIAS_P (mmHg)	med (Q1–Q3)	82.5 (77.5–85.0)	82.0 (79.3–84.5)	0.5	0.905
	MAP (mmHg)	med (Q1–Q3)	99.5 (95.4–103.2)	97.3 (96.0–99.1)	(-) 1.5	0.333
	PR (bpm)	med (Q1–Q3)	83.0 (77.5–91.3)	80.5 (75.0–93.5)	(-) 1.9	0.798
Phase 3	LDF PU (AU)	med (Q1–Q3)	8.3 (7.4–11)	13.0 (9.9–14.7)	37.6	0.025 *
	PSp CRBC (AU)	med (Q1–Q3)	113.4 (99.0–122.3)	101.6 (91.0–119.9)	(-) 6.8	0.386
	SYS_P (mmHg)	med (Q1–Q3)	121.0 (111.5–130.3)	121.0 (116.0–126.3)	2.6	0.513
	DIAS_P (mmHg)	med (Q1–Q3)	77.0 (73.5–82.3)	76.5 (75.0–79.3)	0.8	0.888
	MAP (mmHg)	med (Q1–Q3)	90.0 (87.1–95.3)	90.8 (87.7–93.0)	(-) 0.6	0.677
	PR (bpm)	med (Q1–Q3)	73.5 (69.0–79.8)	74.5 (69.0–77.8)	1.3	0.593

LDF PU, perfusion obtained with laser Doppler flowmetry; PSp CRBC, concentration of red blood cells obtained with polarised spectroscopy; AU, arbitrary units; SYS_P, systolic pressure; DIAS_P, diastolic pressure; MAP, mean arterial pressure; PR, pulse rate; bpm, beat per minute.

## Data Availability

The datasets used and/or analysed during the current study are available from the corresponding author on request.
